# A Comparative Evaluation of Standard and Balloon-Assisted Coiling of Intracranial Aneurysms Based on Neurophysiological Monitoring

**DOI:** 10.3390/jcm11030677

**Published:** 2022-01-28

**Authors:** Stephan Waldeck, René Chapot, Christian von Falck, Matthias F. Froelich, Marc Brockmann, Daniel Overhoff

**Affiliations:** 1 Department of Diagnostic and Interventional Radiology and Neuroradiology, Bundeswehr Central Hospital Koblenz, Rübenacher Straße 170, 56072 Koblenz, Germany; daniel.overhoff@umm.de; 2 Institute of Neuroradiology, University Medical Centre, Johannes Gutenberg University Mainz, Langenbeckstraße 1, 55131 Mainz, Germany; mbrockma@uni-mainz.de; 3 Department of Neuroradiology, Alfried Krupp Krankenhaus, Alfried-Krupp-Strasse 21, 45131 Essen, Germany; rene.chapot@krupp-krankenhaus.de; 4 Institute of Diagnostic and Interventional Radiology, Hannover Medical School, Carl-Neuberg-Straße 1, 30625 Hanover, Germany; vonfalck@icloud.com; 5 Department of Radiology and Nuclear Medicine, University Medical Centre Mannheim, Medical Faculty Mannheim, Heidelberg University, Theodor-Kutzer-Ufer 1-3, 68167 Mannheim, Germany; Matthias.Froelich@umm.de

**Keywords:** balloon-assisted coiling, neurophysiological monitoring, aneurysm coiling

## Abstract

Background and purpose: Intracranial aneurysms are commonly treated with balloon-assisted endovascular coiling because the balloon allows for the control und modulation of wide-necked aneurysms and the coil basket. However, this approach might be associated with a higher complication rate. This retrospective study compared the multimodal results between balloon-assisted coiling of aneurysms (group 1) and coiling without balloon assistance (group 2). Materials and Methods: We included 67 patients with unruptured intracranial aneurysms in this retrospective analysis; acutely ruptured aneurysms were excluded from the analysis. We divided these patients into two groups and evaluated them for symptomatic thromboembolic complications in the course of intracranial aneurysm treatment. All patients had an intrainterventional neurophysiological monitoring (IINM) and a pre- and postinterventional NIH Stoke Scale (NIHSS) survey and MR imaging. Multiple logistic regression was used to assess whether balloon-assisted coiling increased the rate of thromboembolic complications. Periprocedural aneurysm hemorrhage did not occur in any of the cases. Results: We detected no statistically significant difference in rates of neurophysiological disturbances (19.5% (group 1) versus 34.6% (group 2); *p* = 0.249). There was no association with age, gender, or aneurysm location. The occurrence of new diffusion-weighted defects was not statistically significantly different (19.5% (group 1) versus 35.0% (group 2); *p* = 0.166). The difference in NIHSS before and after the intervention showed also no statistical significance in both groups (*p* = 0.426). Conclusion: The use of balloon-assisted coiling did not increase the rate of neurological disturbances during endovascular coiling. MR imaging and NIHSS survey also showed no increased risk of embolization from balloon-assisted aneurysm coiling. IINM is a central aspect of care during endovascular coiling as it can substantially decrease morbidity.

## 1. Introduction

The treatment of intracranial vascular diseases with minimally invasive endovascular techniques has made significant advances in the past years [1,2,3,4]. In most hospitals, endovascular coiling represents the most common therapy for aneurysms [5,6]. With endovascular coil embolization, an aneurysm is treated by filling it with micro coils that promote clotting and close off the aneurysm in order to minimize the risk of rupturing. Through a steerable microcatheter inserted into the bloodstream, the coils accomplish from the inside what a surgical clip would accomplish from the outside: they prevent blood from flowing into the aneurysm but allow blood to flow freely through the normal arteries [7,8,9,10].

Interventional neuroradiologists established significant improvements to the endovascular treatment of intracranial aneurysms, such as balloon- and stent-assisted coiling of wide-necked aneurysms [11,12]. In wide-necked intracranial aneurysms, some coils may protrude out of the aneurysm neck after placement and narrow the parent artery. In order for this adverse event to be prevented, a temporary balloon is inflated to push the coils back into the aneurysm [13]. Despite the fact that these techniques have enabled the treatment of aneurysms that otherwise would have required open neurosurgical clipping, the early balloon-assisted coiling was associated with an increase in the rate of thrombus formation and thromboembolic events [14,15]. An early detection of thromboembolic events would, therefore, be of high, even life-saving, interest.

Intrainterventional neurophysiological monitoring (IINM) during endovascular treatment is a non-stop assessment of the neurological function with the aim of minimizing thromboembolic events through the identification of real-time insults to neurological structures, such that intervention can be initiated to avoid or mitigate resulting injury. IINM uses a combination of diverse tests to gain insights into the functionality of the nervous system. The electrophysiological tests comprise somatosensory evoked potentials (SSEPs), which stimulate peripheral nerves while recording over the scalp and transcranial motor evoked potentials (TcMEPs), where the corticospinal pathways are stimulated electrically and neurogenic and potentials are recorded.

This retrospective study aimed to report our results from intrainterventional neurophysiological monitoring while treating intracranial aneurysms with balloon-assisted coiling (group 1) versus coiling without balloon-assistance (group 2). The incidence of detected neurological disturbances was compared in both groups. These results were additionally compared with preinterventional and postinterventional MR imaging and clinical NIH Stroke Scale (NIHSS) survey.

## 2. Materials and Methods

### 2.1. Patients

The single-center study cohort included 103 patients with endovascular treatment of intracranial aneurysms between May 2013 and February 2018 in a maximum care hospital. Patients treated with stent-assisted coiling were excluded from the analysis. We excluded also ruptured aneurysm treatments. All patients in this study were previously discussed in an interdisciplinary neurovascular conference (neuroradiology, neurology, neurosurgery) before the procedure. On the basis of the access route and based on the feasibility of simple coiling, we decided the use of balloon support pre-interventionally.

Coiling with balloon assistance (group 1): Balloon-assisted coiling was used in 41/67 (61.2%) patients.

Coiling without balloon assistance (group 2): The patients in this group 26/67 (38.8%) were treated with coiling without balloon assistance.

Treatment regimen of both groups:

Pre-interventional MRI imaging was available for both patient groups. Additionally, we performed a clinical NIHSS survey.

Peri-interventional: both groups were subjected to IINM.

Post-interventional: the NIHSS was raised again. MRI imaging was performed the following day.

The basic characteristics of the study population and the locations of the aneurysms can be found in Table 1.

### 2.2. Methods

#### 2.2.1. Endovascular Coiling

All interventions in both groups were performed by using transfemoral arterial access with the patient under general anesthesia. After obtaining arterial access, we routinely administered an initial intravenous bolus of 2500-U heparin.

Coiling with balloon assistance (group 1):

In accordance with current guidelines, the premedication (loading) with aspirin (500 mg) and clopidogrel (300 mg) was administered the day before intervention. We routinely used 8F guiding catheters and placed both the microcatheter and balloon catheter coaxially through the guiding sheath. The balloon length (4–12 mm) and microcatheter tip angulation (45/90 degree) were selected on the basis of neck size and aneurysm location. The deflated balloon catheter was placed across the neck of the aneurysm. If prolapse of the coil was demonstrated during the procedure, the remodeling balloon was inflated to provide secure placement of the coil in the aneurysm without compromise of the parent vessel lumen. For those coils that were placed with the balloon inflated, we would deflate the balloon under fluoroscopic guidance to ensure that there was no migration of the coil before detachment.

Coiling without balloon assistance (group 2):

We routinely used 7F guiding catheters and placed the microcatheter coaxially through the guiding sheath. For all interventions in this group, we used a microcatheter with a 45 degree tip angulation. After complete packing of the coil basket in the aneurysm, we removed the microcatheter under fluoroscopic guidance to ensure that there was no migration of the coil.

#### 2.2.2. Diffusion Weighted Magnetic Resonance Imaging (DWI-MR)

One day after the endovascular coiling intervention, both patient groups were subjected to MRI imaging in order to detect thromboembolic events. A 3 Tesla MRI scanner (Magnetom Skyra; Siemens Healthcare GmbH, Erlangen, Germany) was used. Two experienced neuroradiologists compared the image findings with the pre-interventional MR imaging, and post-treatment DWI-MR image changes were counted and assigned to the related vascular supply area. The lesion loads were categorized into two subgroups:

1. Lesions related to the vascular supply area of the aneurysm.
2. Lesions not related to the vascular supply area of the aneurysm.

#### 2.2.3. Neurophysiological Monitoring

For the IINM in both groups, the ISIS IOM System was used (inomed Medizintechnik GmbH, Emmendingen, Germany). During treatment, IINM electrodes were placed, and baseline SSEPs and TcMEPs were recorded. Changes in SSEPs/TcMEPs hinted thromboembolic events such as ischemic stroke. The changes in SSEPs/TcMEPs were recorded as “neurophysiological disturbances”.

#### 2.2.4. NIHS Score Survey

The NIHSS is a 42 point impairment scale that uses a standardized variety of clinicians in a relatively short time to reveal neurological symptoms and signs in patients with stroke. The clinical neurological examination and the NIHSS score derived from it was collected in a standardized way with a neurological questionnaire pre- and postinterventional in the same way for both groups.

#### 2.2.5. Statistics

Statistical analyses were performed with IBM SPSS (version 23; International Business Machines Corp., Armonk, NY, USA). For descriptive statistics, mean values and standard deviations were calculated. Pearson’s chi-squared test was applied to sets of unpaired categorical data to evaluate the likelihood that any observed difference between the sets was due to chance. Mann–Whitney *U*-test was used to analyze numerical data. The relationship between neurophysiological disturbances as dependent variable and age, gender, aneurysm location, and endovascular coiling with or without balloon assistance as independent variables was determined by using logistic regression.

## 3. Results

The mean age of patients in our study cohort was 62.4 ± 15.7 years. A total of 33 (49.3%) of all patients were male. In 12 out of 33 male (36.4%) and in 5 out of 34 female patients, neurophysiological disturbances were detected peri-interventionally (*p* = 0.053; Table 2).

Although male patients were more likely to suffer from neurophysiological disturbances in both groups, gender was not associated with the occurrence of a neurophysiological disturbance (*p* = 0.087; Table 3). In addition, neither the age of the patient, nor the anatomical localization of the aneurysm (*p* = 0.630; Table 2) exhibited significant influences on the occurrence of neurophysiological disturbances.

Coiling with balloon assistance (group 1):

Neurophysiological monitoring (IINM):

In this balloon-assisted coiling group, 8 out of 41 (19.5%) were accompanied by neurophysiological disturbances during the intervention (*p* = 0.249; Table 2). The anatomical localization was not associated with the occurrence of a neurophysiological disturbance. The balloon-assisted vessel occlusion time (baVOT) ranged for 45 s up to 425 s. Perceptible changes in neurophysiological monitoring during baVOT ranged from 128 s up to 362 s.

**Figure 1:**
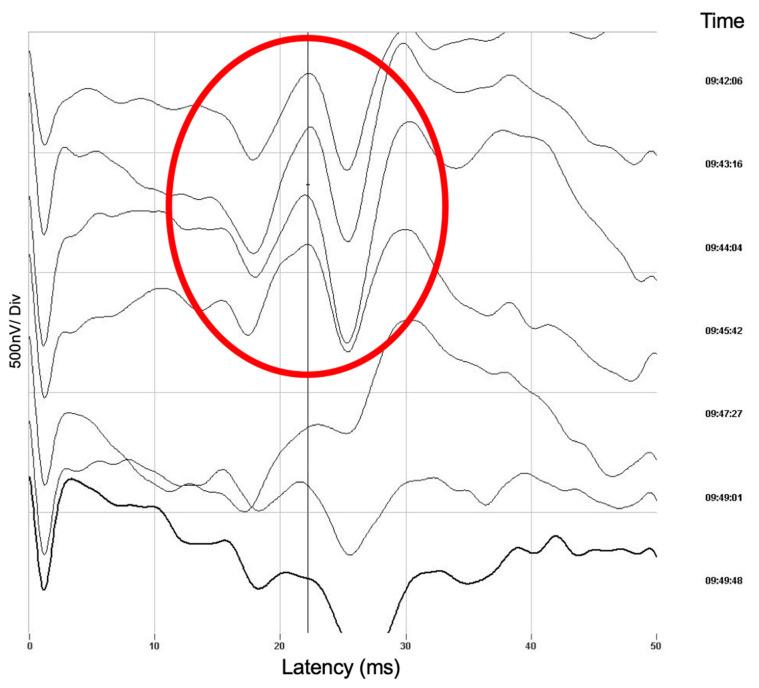
Neurophysiological monitoring during the intervention (IINM). Sudden changes in the evoked potentials as an indication of an M2 occlusion of the middle cerebral artery. These changes occurred after balloon deflation and a resulting coil protrusion.

**Figure 2:**
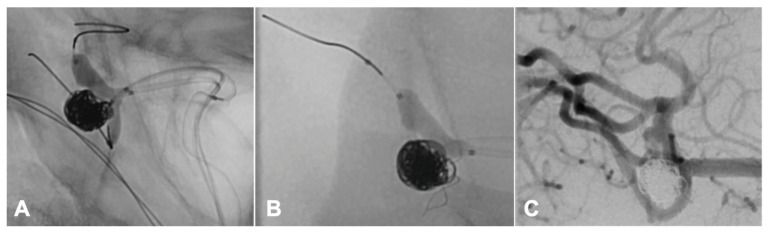
Peri-interventional stroke management. Vessel occlusion due to a coil protrusion into the middle cerebral artery (M2). This coil protrusion increased during the following balloon deflation with subsequent resulting IINM changes (**A**). Positioning a stent through the balloon catheter for coil stabilization (**B**). Regular flow in the DSA control (**C**).

In one patient, who was treated with balloon-assisted coiling and surveilled with intra-operative neurophysiological monitoring, we detected a peri-interventional stroke with neurophysiological monitoring during the intervention (Figure 1), which we could treat immediately by placing a stent (Figure 2).

### 3.1. Diffusion Weighted Magnetic Resonance Imaging

Microembolic lesions appeared in 26% and ranged from 0.32 ± 0.76 per patient in the related subgroup up to 0.10 ± 0.30 per patient in the not related subgroup.

In one patient, who was treated with balloon-assisted coiling and neurophysiological monitoring, we detected a peri-interventional minor stroke on DWI-MR images on the day after the intervention, which has already been indexed by the IINM (Figure 1).

NIHSS Score Survey:

In the balloon-assisted coiling group 1, the patient with the peri-interventional minor stroke had a weakness in the left arm, which regressed after 1 day. This resulted in an NIHSS increase of 1 point. The entire clinical NIHSS before and after the intervention in group 1 showed a non-statistically significant difference (*p* = 0.426; Table 4).

Coiling without balloon assistance (group 2):

Neurophysiological monitoring:

In this standard non-assisted coiling group, 9 out of 26 (34.6%) were accompanied by neurophysiological disturbances during the intervention (*p* = 0.249; Table 2). The anatomical localization was not associated with the occurrence of a neurophysiological disturbance.

Diffusion-weighted Magnetic resonance imaging:

Microembolic lesions in group 2 (coiling without balloon assistance) appeared in 36% of the patients and ranged from 0.5 ± 0.81 per patient in the related subgroup up to 0.12 ± 0.32 per patient in the not related subgroup in MR DW imaging one day after the intervention.

### 3.2. NIHSS Score Survey

The clinical NIHSS score before and after the intervention showed in group 2 not statistically significant difference (*p* = 0.426; Table 4).

In the guideline compliant DSA control after 6 months, there was no coil dislocation in any case.

## 4. Discussion

In this study, we detected no statistically significant difference in rates of peri-interventional neurophysiological disturbances with IINM between aneurysms that were treated by coil embolization with and without balloon assistance.

These results are in agreement with the results of the post-interventional MRI imaging and the clinical outcome, which was evaluated using the NIHSS. The NIHSS focused on the immediate postinterventional outcome compared to the pre-interventional score result.

These findings are important as they suggest relatively similar safety profiles for patients treated with and without balloon assistance. Our results are in agreement with previous reports that documented no increase in complications related to the use of balloon-assisted coiling [1,16,17]. Alternative endovascular treatment method such as double-stent-assisted coiling techniques in an X, Y, and T configuration are not amenable to endothelialization, which may lead to thromboembolic complications [1,16,17]. Moreover, stent-assisted coiling yields high rates of long-term angiographic occlusion, and thus intracranial stenting should be avoided if possible [1,16,17]. We deduce that the use of a balloon in aneurysm treatment not only adds safety in preventing coil dislocation, but also expands treatment options for wide-necked aneurysms. In accordance with the current guidelines (German S3 guideline on diagnosis, therapy, and follow-up of extracranial carotid stenosis), bivalent platelet inhibition should be used when balloons and, if necessary, intracranial stenting are used [18].

Neurophysiological monitoring is a central tool of intraoperative and intrainterventional care critical for detecting central and peripheral ischemic events. The recognition of potential changes in SSEPs/TcMEPs, which could be caused by intracranial hemorrhage or ischemic stroke, is essential in the avoidance of permanent deficits. In one cause of an intrainterventional minor stroke, we were able to detect the event first in IINM changes. Without neurophysiological monitoring, we would have detected the vascular occlusion later.

Especially with regard to the great temporal variability of the neurophysiological changes in baVOT, our study results indicate that the standardized use of IINM in endovascular aneurysm treatment provides more certainty in the detection of ischemic and thromboembolic events, regardless of whether the aneurysm was treated with or without balloon protection.

Early diffusion-weighted MRI lesions after treatment of unruptured intracranial aneurysms is a recognized procedure. It is used in routine diagnostics both after surgical aneurysm treatment by clipping and after endovascular aneurysm treatment. DWI lesions reliably indicate both clinically apparent and silent thromboembolic events. The DWI lesions are comparable in both procedures. Our results in the frequency of DWI lesions are consistent with recent findings in the literature. Here, the frequency of postinterventional DWI lesions ranged from 23 to 40% [19].

In our study, all postinterventional findings in DWI imaging one day after the intervention were in complete agreement with the IINM (Table 4).

## 5. Study Limitations

Our study has several limitations. First, it is a retrospective single-center study. It cannot be completely ruled out that confounding factors exist between the two groups or that selection bias occurred, although great care was taken in the analysis of the data. Secondly, as our study population included only 67 patients with unruptured intracranial aneurysms, we cannot exclude a type 2 error as explanation for the lack of association.

Our study focused on intra- and periinterventional outcome. The clinical outcome analysis was only measured by the NIHSS. The NIHSS provides limited information on how a minor stroke affects patients in their daily lives (e.g., on a functional and/or emotional level) [20]. Long-term outcome was not the primary target of this study.

Further studies should investigate a longer clinical course of the patient after aneurysm treatment.

We are aware that this finding needs replication in larger aneurysm populations.

## 6. Conclusions

The use of balloon-assisted coiling did not increase the rate of neurophysiological disturbances during endovascular interventions. The results are consistent with postinterventional MRI findings and the clinical outcome.

The combination of balloon-assisted coiling and periinterventional neuromonitoring is thus a procedure that has the potential to increases safety in the treatment of intracranial aneurysms without posing additional risk.

## Figures and Tables

**Table 1:** Basic characteristics of the study populations.

Variable	Coiling with Balloon (Group 1)	Coiling without Balloon (Group 2)
Number of interventions	41	26
Number of male patients	17 (41.5%)	16 (61.5%)
Average age of patients in years (mean ± SD)	62.0 ± 16.8	53.7 ± 25.1
Location of aneurysm		
Basilar artery	2	2
ICA	0	2
ICA, bifurcation	1	2
ICA, C4	1	1
ICA, C5	1	1
ICA, C6	1	1
ICA, C7	7	4
PICA	5	6
MCA, bifurcation	10	0
ACoA	5	6
PCoA	4	4

SD—standard deviation; ICA—internal carotid artery; PICA—posterior inferior cerebellar artery; MCA—medial cerebral artery; ACoA—anterior communicating artery; PCoA—posterior communicating artery.

**Table 2:** Association of neurophysiological disturbances with gender, coiling approach, and anatomical location of the aneurysm.

Variable	Manifestation	No Disturbance Detected	Disturbance Detected	*p*-Value
Gender	Male	21	12	0.053
	Female	29	5	
Coiling	Without balloon (group 2)	17	9	0.249
	With balloon (group 1)	33	8	
Anatomical localization	ACoA	11	4	0.630
	ICA	1	1	
	Basilar artery	1	2	
	ICA, bifurcation	2	1	
	MCA, bifurcation	9	4	
	ACoP	7	1	
	PICA	8	1	
	ICA, C4	2	0	
	ICA, C5	2	0	
	ICA, C6	2	0	
	ICA, C7	5	3	

Multiple logistic regression for association of neurophysiological disturbances with gender, coiling approach, and anatomical location of the aneurysm. *p* ≤ 0.05 indicates statistical significance; ACoA—anterior communicating artery, ICA—internal carotid artery; MCA—medial cerebral artery, ACoP—posterior communicating artery, PICA—posterior inferior cerebellar artery.

**Table 3:** Multiple logistic regression on the occurrence of neurological disturbances.

Variable	Odds Ratio (95% Confidence Interval)	*p*-Value
Gender	0.341 (0.100–1.167)	0.087
Age	0.997 (0.981–1.013)	0.737
Anatomical localization of aneurysm	0.978 (0.815–1.173)	0.811
Coiling with ballon	0.545 (0.174–1.708)	0.298

*p* ≤ 0.05 indicates statistical significance.

**Table 4:** Evaluated results of the study populations.

Variable	Coiling with Balloon	Coiling without Balloon	*p*-Value
Neuromonitoring detect	8 (19.5%)	9 (35.0%)	0.166
Lesion load-related	0.32 ± 0.76	0.5 ± 0.81	0.190
Lesion load not related	0.10 ± 0.30	0.12 ± 0.32	0.818
MRI-DWI	8 (19.5%)	9 (35.0%)	0.166
NIHSS difference	0.02	0.00	0.426
Event	1 (2.4%)	0 (0%)	0.422

Categorial data are given as absolute numbers and percentage. Numerical data are given as mean and SD. *p* ≤ 0.05 indicates statistical significance.

## Data Availability

The data presented in this study are available on request from the corresponding author. The data are not publicly available due to ethic restrictions.

## Abbreviations

baVOT	balloon-assisted vessel occlusion time
DWI-MR	diffusion-weighted magnetic resonance
IINM	intrainterventional neurophysiological monitoring
NIHSS	NIH Stroke Scale
SSEPs	somatosensory evoked potentials
TcMEPs	transcranial motor evoked potentials
